# Treatment and rehabilitation outcomes of children affected with nodding syndrome in Northern Uganda: a descriptive case series

**DOI:** 10.11604/pamj.2018.29.228.13627

**Published:** 2018-04-26

**Authors:** Suzanne Gazda, David Lagoro Kitara

**Affiliations:** 1 Hope for Humans (HfH), San Antonio, Texas, USA; 2 Gulu University, Faculty of Medicine, Department of Surgery, Gulu, Uganda

**Keywords:** Nodding syndrome, Hope for HumaNs (HfH), food rehabilitation, growth and improved quality of life

## Abstract

**Introduction:**

Nodding Syndrome (NS) is a neurological disorder affecting children 5-15 years at onset in East Africa. A major criterion for diagnosis is atonic seizure with dorso-ventral “nodding” of the head. Comorbidities include psychological and behavioral abnormalities, malnutrition, cognitive decline, school dropout and other seizure types. We aimed to describe the presentations and rehabilitation outcomes of NS children at Hope for HumaNs (HfH) centre in Gulu from September 2012 to October 2013.

**Methods:**

Data was obtained from a retrospective review of 32 NS children's medical records at HfH center. Ethical approval was obtained from Gulu University IRB. Data analysis was conducted using WHO AnthroPlus, SPSS and Excel software.

**Results:**

Growth statistics showed steady improvement over time using local nutrition and multivitamin supplementation. Severe and moderate stunting was reduced from a combined total of 54.8% to 7.7% and 12.8% respectively. Severe and moderate wasting was reduced from 29.1% to 2.6% and 5.1% respectively. Three groups of NS children were identified and compared in the review; Low seizure occurrence averaging <2 seizures/month (28.1%); Moderate averaging 2-4 seizures/month (34.4%) and High averaging >4 seizures/month (37.5%).

**Conclusion:**

NS is a neurological disorder of unknown etiology. Treatment with regular high quality local nutrition, multivitamin supplementation, anti-seizures, regular follow up and illness prevention; children's seizures can be reduced or stopped completely. The debilitating malnutrition and stunting of NS children in Uganda could be partially independent of the syndrome but attributable to poor nutrition. NS as observed is not “invariably fatal” but rather a treatable neurological disorder.

## Introduction

"Nodding Syndrome (NS) should be considered a critical and pervasive threat to human security in the affected communities of Northern Uganda, because it exacerbates vulnerability of people in the process of recovering from violent conflict" [1]. Nodding Syndrome typically affects young children that are subject to civil disruption, internal displacement, food insecurity, malnutrition and nematode Onchocherca Volvulus (OV) infection [2]. This neurological disorder of unknown etiology affects thousands of children in Northern Uganda, South Sudan and Southern Tanzania [3]. The burden it places upon affected communities is multifaceted, ranging from physical and mental health decline of an individual child to increasing health disparities of an entire community [3]. The economic, psychosocial and health structures are all affected by this epidemic [3]. Government interventions were to include; In and Outpatient screening and treatment along with subsequent follow-up care, psychological and social support for families, rehabilitation services with occupational, physical, speech and language therapy to mitigate loss of function and prevent further disability [4]. However, there have been variable reports on the occurrence and results of these interventions [5-7]. This is likely due to the already pervasive weakening of these health structures in the area from years of internal conflict and a slow post-conflict recovery [1]. We conducted this study with the aim of exploring and presenting the clinical findings and reporting treatment and rehabilitation outcomes of NS children at the Hope for HumaNs (HfH) rehabilitation centre that was set up in Odek Sub county, Gulu district, Northern Uganda in 2012 which showed that NS was not “invariably fatal” but rather a treatable neurological disorder.

## Methods

**Study design**: This was a retrospective review of medical records of NS children who were under rehabilitation at the HfH care centre in Northern Uganda. These NS children were assessed before enrolment into care by a multidisciplinary team constituted by Ugandan Ministry of Health (MOH), Gulu District Health Department and Gulu University in 2012. The admission criteria were based on WHO epidemiological and surveillance case definition of probable Nodding Syndrome [8-11].

**Figure 1:**
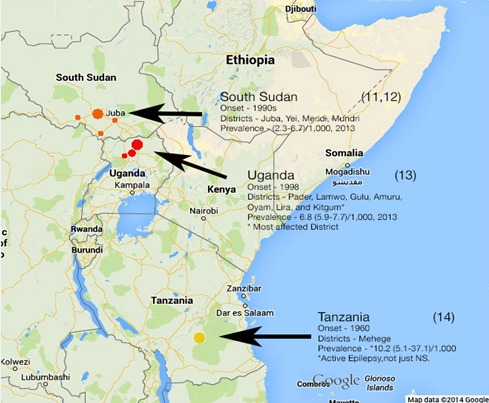
Map of Nodding syndrome occurrence in East Africa

**Study site**: This study was conducted at a Non-Governmental Organization (NGO), Hope for Humans (HfH) care centre for rehabilitation of NS children at Aromowang lobo (Figure 1). This centre was built in 2012 as a private initiative by two American founders from Texas, USA with the help of a Ugandan professor/clinician, the author (DLK). It's a facility with classrooms for teaching and learning; a medical clinic for treatment, refectory and cooking place for food rehabilitation, a play field for soccer; a piggery for livelihood project and medical staff residence. There was a daily schedule of activities for NS children beginning with travel from home, registration, administration of medication, physical exercises, feeding, bathing, personal hygiene, and physiotherapy [9, 12].

**The study population**: The study population was originally 42 NS children but one child dropped out before the end of the study and two with poor attendance were excluded. Poor attendance was defined as any child missing more than 50% of their monthly seizure history report for two consecutive or nonconsecutive months between September 2012 and October 2013. On average, the attendance at the center was 75.4% (95% CI ± 7.4%). Six NS children were excluded due to lack of major criteria of head nodding history; one was excluded because the child was less than 3 years at diagnosis, thus a total of 32 records were retrieved and used for this analysis. Importantly, NS children attended care at the HfH centre from Monday to Saturday (8:00am-4:00pm EST). Gulu District Health Department managed all changes in medications, dosing and frequency of dosing for NS children together with other interventions such as vaccinations and treatment of illnesses. Nurses at the center provided wound care, medicine administration and documentation of growth, illnesses and seizure frequencies. A daily seizure record was kept for each NS child. Seizures that occurred over night and at home or on Sundays were reported to staff during morning roll call the next day and recorded in each child's seizure diary. All nursing and teaching staffs at the HfH center were trained in reporting head nodding, generalized and partial seizures, along with absence seizures/staring spells. All seizure reports were reviewed and documented by the school nurse who lived at the center throughout the week.

**Data sources**: We used the Ugandan Ministry of Health Head Nodding Syndrome Health Facility Case Form (HFCF).The Nodding Syndrome Task Force (NSTF) screened these children in July and August 2012. Growth statistics were recorded on admission of NS children to the center and seizure documentations were made consistent from September 2012 to October 2013. All NS children at the center were provided with regular meals, multivitamin supplementation and anti seizure medications as per the prescriptions.

**Ethical consideration**: This study was approved by Gulu University IRB (GUIRC) (GU/IRC/03/09/13). Parents/Guardians of NS children gave informed consent for the participants' review but for those above 14 years but below 18 years, assent was obtained. In addition, the study was conducted in accordance with good clinical practice and confidentiality of NS medical records was maintained with the patients' identity anonymized and only accessible to the Principal Investigator. We obtained informed consent from parents/guardians for this information to be published in medical journals for the wider scientific community.

**Statistical analysis**: Data was obtained from three major document files; The Ugandan MOH Nodding Syndrome Health Facility Case Form (HFCF); HfH Seizure Diaries, and the children's medical history books. Growth metrics were converted to z-scores using WHO AnthroPlus 3.2 software and then compiled with data from the initial assessment on the MOH NS case form and daily seizure activity into Microsoft excel (for Mac 2011-Version 14.2.4(120824)) statistical tests and were computed by IBM SPSS statistics version 24. Descriptive statistics were used to present the information obtained and where explicitly required, we used bivariable analysis.

## Results

**Figure 2:**
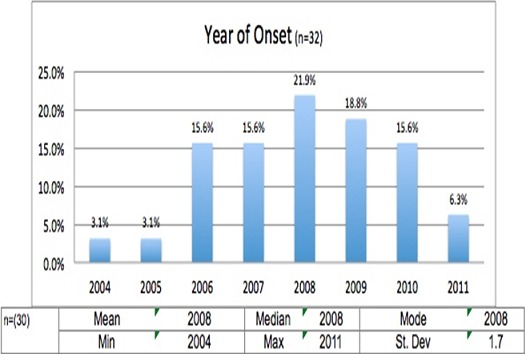
Year of nodding onset among the 32 NS children studied at HfH Centre

The demographic information in the NS children's admission files mirrored previous studies [4, 8-10, 13-15]. The study was conducted in Odek, Gulu, Northern Uganda; one of sites were NS has been identified by WHO in East Africa i.e South Sudan, Southern Tanzania and Northern Uganda (Figure 1). The study population (n = 32) composed of 18 males and 14 females, between the ages of 8 and 15 years with a mean age of 12.7 SD + 1.6 years (Table 1). The year of nodding onset ranged from 2004 to 2011, with the model year of 2008/2009 (Figure 2). Nodding Syndrome children's medical records showed great variability in severity of the illness (perhaps spectrum occurrence) including psychological effects (Table 2). Some presented with frequent monthly seizures, while others had manageable seizures averaging less than one seizure a month (Table 3).

**Table 1:** Socio-demographic characteristics of the 32 NS children studied at the HfH

Ages (yrs)	Frequency	Percentages (%)
8	1	3.1
9	0	0.0
10	2	6.3
11	3	9.4
12	8	25.0
13	5	15.6
14	11	34.4
15	2	6.3
**Subtotal**	32	100.0
**Villages of the NS children**		
Ajan IDP camp	1	3.1
Ban pii IDP camp	1	3.1
Puranga IDP camp	1	3.1
Ayom IDP camp	2	6.3
Agago IDP camp	1	3.1
Rackoko IDP camp	1	3.1
Aromo IDP camp	1	3.1
Atyang IDP camp	1	3.1
Awere IDP camp	4	12.6
Akoyo IDP camp	19	59.4
**Subtotal**	32	100.0
**Relationship between NS diagnosis in 2012 with school dropout of each NS child**		
Dropped out before NS diagnosis	2	7
Dropped out in the year of NS diagnosis	10	33
Dropped out a year after NS diagnosis	6	20
Dropped out 2 years after NS diagnosis	4	13
Dropped out >2years after NS diagnosis	2	7
Never attended School	5	17
In school currently	1	3
**Subtotal**	30	100

**Table 2:** The psychological findings in 32 NS children at HfH rehabilitation centre

Variables (n=32)	Percentages (%)
Normal Appetite	44.0
Reduced Appetite	39.0
Increased Appetite	17.0
Emotionally feels normal	46.4
Feels sad most of the time	50.0
Feels anxious and fearful most of the time	14.3
Feels excited	14.3
Reports bad dreams	18.0
Reports sleeping too much	18.0
Reports lacking sleep	18.0
Reports normal sleep	46.0
Reports normal thoughts	61.5
Reports worrying most of the time	42.3
Reports thoughts about dying	15.4
Reports thoughts of killing self	23.1

**Table 3:** The descriptive statistics for the nodding episodes of the 32 NS patients

Nodding/month (n=32)	Mean (%)	Median (%)	STD (%)	(95% Confidence Interval) (%)
Reporting 0 nodding episode	45.2	40.6	9.7	(39.3, 51.0)
Reporting 1 nodding episode	12.0	9.4	7.1	(7.7, 16.3)
Reporting 2-4 nodding episodes	28.9	28.1	9.2	(23.3, 34.4)
Reporting >4 nodding episodes	31.3	31.3	10.7	(24.8, 37.7)

STD: Standard Deviation

## Discussion

Recent studies on NS have begun the discussion that although the etiology of Nodding Syndrome may be unknown, it's a treatable neurological disorder that has been demonstrated by the Hope for HumaNs (HfH) centre in Odek, Gulu, Uganda [16, 17]. These studies showed that with symptomatic treatment, substantial clinical and functional improvements are demonstrable and possible in NS children [17].

**Figure 3:**
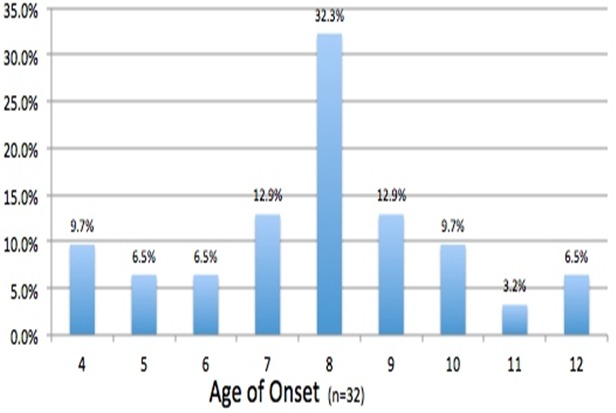
Age of nodding onset among the NS children studied at HfH Centre

**Figure 4:**
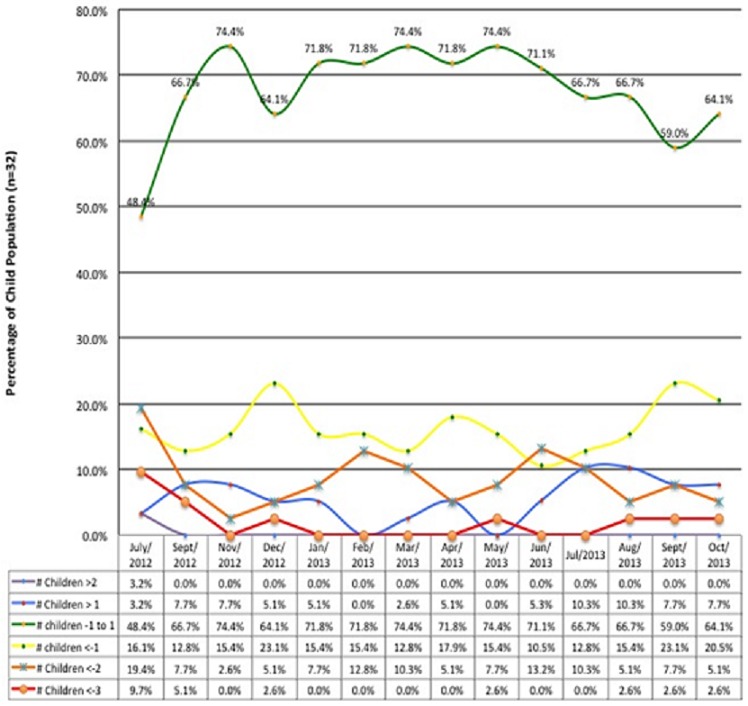
(BMI for Age (z-scores) for the 32 NS children studied at HfH Centre

**Figure 5:**
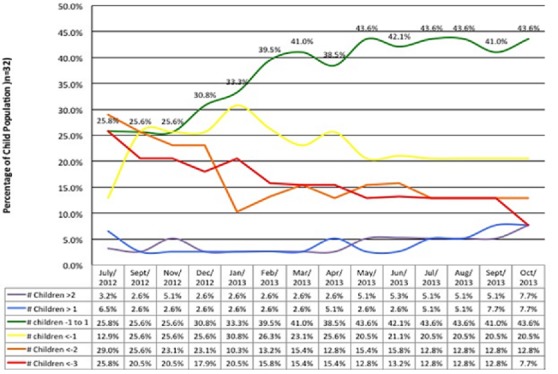
Height for Age (z-scores) for the 32 NS children studied at HfH Care Centre

**The physical and psychological observations in 32 NS children studied at HfH centre**: The psychological variables from HFCF reviewed included; school dropout in close proximity to diagnosis of NS in 2012, behavioral changes, emotional problems, social support and sleep problems (Table 1, Table 2). In addition, the reports showed that 53% of children dropped out of school within a year or one year after nodding onset (Table 1). While some developed seizures simultaneously with head nodding and some never at all; most occurred within 1 to 3 years of initial NS symptoms' onset [14]. Parents of NS children reported that NS children were expressing aggressive behavior in 48.1%; anxiety in 14.3%; depression in 50% and loss of appetite in 39% (Table 2). Furthermore, reports from caregivers observed; epileptic fits in 82.6% and jerking fits in 55.6% of NS children at the time of admission to the HfH center (Table 3). The concerns about risk of self-harm among NS children were reinforced by findings that 23.1% of them reported thoughts of killing self during the period (Table 2). The age of onset of NS among the children peaked at 8 years (32.3%) but there were two semi-peaks at 7 years (12.9%) and 9 years (12.9%) respectively (Figure 3). In addition, it was noted that on admission of NS children at the HfH center in 2012 using WHO criteria for surveillance and epidemiological diagnosis [4], their nutritional status were poor, NS children presenting with moderate to severe wasting was 29.1% and those presenting with moderate to severe stunting was 54.8% (Figure 4, Figure 5). It was observed that after 13-months of regular nutritional, multivitamin supplementation and anti seizure medications; severe wasting was reduced from 9.7% to 2.6% and moderate wasting reduced from 19.7% to 2.6% respectively while severe and moderate stunting was reduced from a combined prevalence of 54.8% to 12.8% and 7.7%, respectively. At the end of this study period, 64.1% of NS children were within normal Body Mass Index (BMI) for age (z-score) ranges (-1 to 1) with 15.4% being above average and 20.5% having mild thinness (Figure 4). The severely low BMI for age (z-scores) that remained unimproved over the period was for a single 13 year old male who presented with dramatic stunting with an appearance of 6 years old child (Figure 4). In addition, severe and moderate stunting improved substantially from baseline admission figures and by the end of this study, 58% were above or at average height for age, while 20.5% were just below average (Figure 5). One case of moderate to severe stunting remained unimproved despite adequate nutrition and multivitamin supplementation (Figure 5). Furthermore, stunting for the majority of cases was corrected with regular local nutrition and multivitamin supplementation (Figure 5). This could mean potentially that physical wasting and stunting were perhaps due to poor appetite, uncontrolled seizure affecting their eating habits, endemic poverty affecting family resources and preventing access to good nutrition (Table 2).

**Figure 6:**
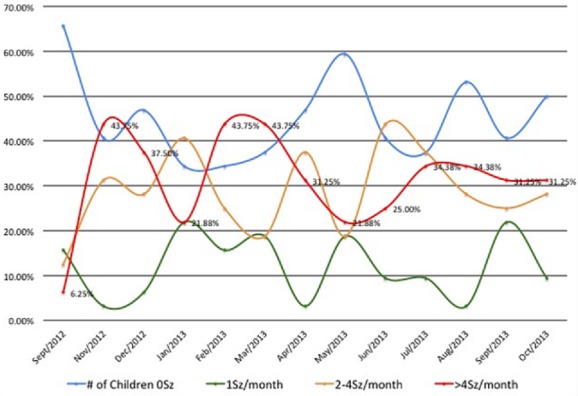
HfH monthly seizure reports for the 32 NS children studied

**Figure 7:**
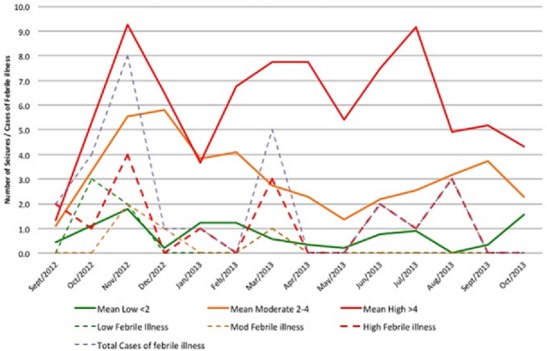
Monthly pattern of seizures with respect to febrile illnesses among NS children

**The descriptive statistics for nodding episodes**: Interestingly, the monthly nodding reports showed that on average, 31.3% of NS children reported more than 4 seizures a month (Table 3). This information helped the facility anticipate the required care and mitigate NS children's risk and provide safety (Table 3). During monitoring, three months of increased seizure activities were observed; November 2012 (43.8%); February 2013 (43.8%); and July 2013 (34.4%) (Figure 6). These peak months correlated in space and time with febrile illnesses and reduced medical interventions to NS children in the community. All three peaks were associated with increases in febrile illnesses and cold weather experienced by the children (Figure 7). The second peak in February 2013 occurred in relationship with the end of outreach clinics in February 2013, when there was lack of funds for fuel to deliver medications to the clinics [6]. The increases was maintained throughout March during a period of reported increased febrile illnesses and then decreased to 21.9% in May. The third peak in July 2013 occurred when the HfH center had 6 cases of febrile illnesses in close proximity (Figure 7). These three peaks declined over time (43.8%, 43.8%, and 34.4%) respectively. This could have perhaps been due to improved coping mechanisms by NS community or that the febrile illnesses were spread over months or/and improved Ugandan Ministry of Health intervention to deficits in care. Overall NS children averaged 3.6 seizures/month during the period (95% CI ± 1.24). Three distinct groups of NS children were observed (Low, moderate and high seizure frequencies). Low was defined as NS children averaging less than 2 seizures a month (28.1%); moderate as 2-4 seizures a month (34.4%), and High as greater than 4 seizures a month (37.5%).

**The treatment regimen for the 32 NS children**: Observing the duration of therapy for each group; NS children on VPA monotherapy were for 9 months (95% CI ± 1.8) with a mean of 23.6mg/kg/day (95% CI ± 3.4) (Table 4). Overall, these NS Children averaged 3.2 seizures a month for the 13-month period (95%CI ± 1.3). Children on VPA+CBZ combined were on VPA for an average 7 months (95% CI ± 2.0) and CBZ for an average 10 months (95%CI ± 4.0), with a mean of 21.9mg/kg/day (95%CI ± 3.9) and 7.5mg/kg/day (95%CI ± 1.8) respectively. The group taking a combined VPA+CBZ averaged 4.3 seizures per month (95% CI ± 1.5) for the period. It was observed that only a single case of NS child taking CBZ only (11.7mg/kg/day) had an average 0.6 seizures per month while the other children on CBZ monotherapy were more likely to have a higher monthly average seizure frequency compared to others. In the high seizure group; 60% of children (n = 10) were on VPA + CBZ, while the moderate and Low groups were 50% (n = 10) and 40% (n = 7) respectively. The high seizure group were more likely to be females (80%, n = 12) and had more cases of febrile illnesses (18 cases, 1.5 per child) reported as compared to low (6 cases, 0.8 per child) and moderate categories (3 cases or 0.3 per child). Male NS children were also more likely to have a higher average seizure free months (4.3 months, 95% CI ± 1.8) compared to females (2.8 months, 95% CI ± 1.8). It was not yet clear the reasons for these differential observations. These authors suggest that more studies should be conducted to understand this further.

**Table 4:** The treatment regimens for the 32 NS children studied at HfH care centre

AED treatment	Mean	Median	STD	(95% CI)	Mean number of Seizures (Sz)
VPA (n = 14)	23.6	21.9	6.1	20.2, 26.9	3.2
VPA + CBZ (n = 14)	22.0	21.9		17.9, 26.1	4.3
CBZ (n = 1)	11.6				0.6

VPA= Sodium Valproate; CBZ=Carbamazepine; STD=Standard Deviation; CI= Confidence Interval;

Sz= Average number of seizures; AED = Anti-epileptic drugs; HfH= Hope for HumaNs.

The majority of NS children by the end of this observational period were on Sodium Valproate (48.3%), Carbamazepine (CBZ) 1/32(3.4%) or both (48.3%).

**Hope for HumaNs (HfH) monthly seizure reports**: This is shown in Figure 6 and it indicates that there was a general reduction in frequency of nodding among NS children reviewed. The majority of NS children demonstrated significant reduction in the frequencies of nodding. Perhaps the regular dosing with anti-seizure medications, food supplementation and better care at the HfH centre could be the major contributing factor. Notably a great number of children remained seizure free for several months.

**Monthly pattern of seizures with respect to febrile illnesses among NS children**: The overall pattern of febrile illnesses (Violet dotted lines) in Figure 7 show patterns corresponding fairly with the high mean seizures which peaked in November 2012 and two semi peaks in March 2013 and August 2013. With this observation showing febrile illnesses and seizure frequencies matching closely, it could be suggested that seizure frequencies could perhaps be related to the frequencies of febrile illnesses which we earlier on suggested that NS is perhaps a metabolic disorder which is precipitated by high demands for energy requirements that can be experienced during febrile illnesses [2, 14, 15]. It is well documented in previous studies that cold weather, exposure to cold water and the sight and eating of local foods stimulate nodding in NS children [2, 14, 15] however, there also exist the potential that febrile illnesses could represent another of several antecedent stimuli. When comparing the timing of illness in the children's medical books to the children's seizure diaries, NS children with more episodes of febrile illnesses showed higher monthly seizure averages (Figure 6, Figure 7). In addition, one study had reported seizures being induced by eating hot foods [15]. However, these authors suggest that further studies are needed to assess if these seizures were solely due to febrile illnesses, or rapid changes in core temperatures or changes in arousal states of NS children (Figure 6 & Figure 7).

Interestingly, this retrospective review has expanded knowledge on seizure control and its changes over time in NS children. Malnutrition resulting into stunting and wasting has also been shown to improve with treatment and food rehabilitation with only a small population of NS children remaining severely stunted (Figure 4, Figure 5). This small group is likely to represent the advanced stages of the syndrome and late intervention with irreversible stunting and perhaps possible changes in growth hormone functioning [18]. The reversibility of these symptoms is likely depended on the timing of interventions and the intrinsic problems of each individual NS child. In addition, NS children who presented with more frequent episodes of febrile illnesses appeared to be at higher risk of frequent seizure activity as compared to those with less (Figure 6, Figure 7). This could perhaps be due to the fever itself, the body's immune response to illnesses or changes in arousal state [15] or environmental factors leading to these illnesses. In addition, female NS children appeared to carry a heavier seizure burden over the observational period with a mean of 4.7 seizures a month compared to males with an average 2.7 seizures a month. It is unknown whether this is due to gender differences versus social or environmental factors predisposing young NS females to illnesses and seizure precipitating events. In addition, the cyclic pattern of seizure frequency is also worth noting since cross-sectional studies could be impacted by this kind of fluctuations. Therefore, body temperature and seasonal impact on seizure frequency could bear potentials for further investigations (Figure 7). Additionally, reports on variability in health intervention; lack of funding, personal experiences, availability of food, funds and disruptions of medication supply chains all have potential to substantially inhibit care and patients' safety and are points of concern [5, 19, 20]. Medication shortages and anecdotal reports in the media of resulting deaths raises warnings of a weak health infrastructure and a compromised care for NS affected families [21]. Finally, as information on effective treatments grows, funding together with appropriate interventions need to follow this well documented observation so that children with Nodding syndrome could receive better quality life.

**Strengths and limitations of this study**: Potential sources of error could exist in the documentation of the various seizure types. Nodding Syndrome seizures can be similar to other convulsive disorders. It is worth noting that due to differences in the level of staff training at the HfH care center, seizure types along with duration of the events may have not been recorded consistently. This could introduce potential errors as other convulsive disorder types could appear as Nodding Syndrome. To minimize this risk, children were screened by the Nodding Syndrome Task Force before admission into care and were reviewed for the presence of head nodding by the Gulu District Health Department and the Nodding Syndrome Task Force experts. Children lacking this major criterion upon admission were not included in this study. Another potential source of error was parental reports of seizures while NS children were at home over night, or on absentee days, and/or on their days off or on Sunday when the children were not at the HfH care centre. In addition, NS children's memory of seizure post-occurrence was likely to be poor. Furthermore, seizures unobserved by a parent or caretakers were potential sources of errors. However, in spite of all these potential sources of errors, the consistency by which the care givers made their reports at different times to health workers at the Government Health Centres corroborated that the information given to the HfH centre was true and accurate.

## Conclusion

Nodding Syndrome is a childhood neurological disorder of unknown etiology. Treatment and rehabilitation with regular high quality local nutrition, multivitamin supplementation, anti-epileptics, regular follow up and illness prevention; NS children's seizures can be reduced or stopped completely. The debilitating malnutrition and stunting of NS children in Northern Uganda could be partially independent of the syndrome but attributable to poor nutrition. NS as observed is not “invariably fatal” but rather a treatable neurological disorder.

### What is known about this topic

- Nodding syndrome is a childhood neurological disorder only identified in East Africa;
- Nodding syndrome is associated with cognitive decline and school dropout;
- Nodding syndrome children were internally displaced before developing the syndrome.

### What this study adds

- Nodding syndrome is a treatable neurological disorder;
- Febrile illnesses and increased in core body temperature seems to be related to increased frequencies of nodding episodes;
- The nodding episodes can be controlled completely and NS children can return to school but remains with residual emotional and perceptual difficulties.

## Competing interests

All authors declare no competing interests.
